# Identification of an Epithelial-Mesenchymal Transition-Related Long Non-coding RNA Prognostic Signature to Determine the Prognosis and Drug Treatment of Hepatocellular Carcinoma Patients

**DOI:** 10.3389/fmed.2022.850343

**Published:** 2022-05-24

**Authors:** Shenglan Huang, Dan Li, Lingling Zhuang, Jian Zhang, Jianbing Wu

**Affiliations:** ^1^Department of Oncology, The Second Affiliated Hospital of Nanchang University, Nanchang, China; ^2^Jiangxi Key Laboratory of Clinical and Translational Cancer Research, Nanchang, China; ^3^Department of Gynaecology, The Second Affiliated Hospital of Nanchang University, Nanchang, China

**Keywords:** hepatocellular carcinoma, epithelial-mesenchymal transition, long non-coding RNA, prognostic signature, nomogram

## Abstract

**Introduction:**

Hepatocellular carcinoma (HCC) is one of the most common malignant tumors with poor prognosis. Epithelial–mesenchymal transition (EMT) is crucial for cancer progression and metastasis. Thus, we aimed to construct an EMT-related lncRNA signature for predicting the prognosis of HCC patients.

**Methods:**

Cox regression analysis and LASSO regression method were used to build an EMT-related lncRNAs risk signature based on TCGA database. Kaplan-Meier survival analysis was conducted to compare the overall survival (OS) in different risk groups. ROC curves and Cox proportional-hazards analysis were performed to evaluate the performance of the risk signature. RT-qPCR was conducted in HCC cell lines and tissue samples to detect the expression of some lncRNAs in this risk model. Furthermore, a nomogram involving the risk score and clinicopathological features was built and validated with calibration curves and ROC curves. In addition, we explored the association between risk signature and tumor immunity, somatic mutations status, and drugs sensitivity.

**Results:**

Twelve EMT-related lncRNAs were obtained to construct the prognostic risk signature for patients with HCC. The Kaplan-Meier curve analysis revealed that patients in the high-risk group had worse overall survival (OS) than those in low-risk group. ROC curves and Cox regression analysis suggested the risk signature could predict HCC survival exactly and independently. The prognostic value of the risk model was confirmed in the testing and entire groups. We also found AC099850.3 and AC092171.2 were highly expressed in HCC cells and HCC tissues. The nomogram could accurately predict survival probability of HCC patients. Gene set enrichment analysis (GSEA) and gene ontology (GO) analysis showed that cancer-related pathways and cell division activity were enriched in high-risk group. The SNPs showed that the prevalence of TP53 mutations was significantly different between high- and low-risk groups; the TP53 mutations and the high TMB were both associated with a worse prognosis in patients with HCC. We also observed widely associations between risk signature and drugs sensitivity in HCC.

**Conclusion:**

A novel EMT-related lncRNAs risk signature, including 12 lncRNAs, was established and identified in patients with HCC, which can accurately predict the prognosis of HCC patients and may be used to guide individualized treatment in the clinical practice.

## Introduction

Hepatocellular carcinoma (HCC) is one of the most common aggressive tumors that ranks as the fourth leading cause of cancer-related mortality worldwide (1). Despite the proven efficacies of several treatment methods, such as surgical resection, liver transplantation, ablation therapy, radiotherapy, targeted therapy, systemic chemotherapy, and immunotherapy, the prognosis remains unsatisfactory. According to estimates from the World Health Organization, more than 1 million patients will die from HCC by 2030, with the 5-year survival rate being no more than 18% (2, 3). Such poor survival rates are mainly due to lack of reliable predictive markers. Therefore, there is an urgent need to identify highly sensitive and specific prognostic biomarkers for predicting the clinical outcomes of HCC patients.

Epithelial-mesenchymal transition (EMT) is a process that leads to cell morphology and function changes, which is characterized by the dissolution of cell-cell junctions, synthesis of extracellular matrix, increasing cell motility that consequently result in the cells gaining invasive and metastatic properties (4, 5). Numerous studies have shown that EMT plays a vital role in the development and progression of HCC (6, 7). For example, transforming growth factor-β (TGF-β), one of the most potent inducers of EMT, is correlated with carcinogenesis, invasion and metastasis in HCC (8). The EMT-transformed HCC cells stimulated with TGF-β were resistant to sorafenib-induced apoptosis (9). Moreover, mounting studies have shown that EMT contributes to immunosuppression, and EMT process is associated with chemotherapy and immunotherapy resistance (10). Therefore, EMT-related genes may be used as novel and ideal prognostic predictors and therapeutic targets for the patients with HCC.

Long non-coding RNAs (lncRNAs), which account for 80–90% of all ncRNAs, structurally contain more than 200 nucleotides. It has been wildly applied in early diagnosis and prognosis biomarkers of various tumors (11, 12). Accumulating evidences suggested that lncRNAs are involved in regulating EMT processes in a variety of tumors, including HCC (13); such as lncRNA-POIR (14), lncRNA-AB209371 (15), lncRNA CRNDE (16), lncRNA SNHG3 (17), and lncRNA LOC554202 (18), have been reported to promote the epithelial-mesenchymal transition, accelerate the tumor metastasis, and suppress the drug sensitivity of HCC cells. Conversely, lncRNA CASC2 and lncRNA miR503HG inhibit EMT and exert anti-metastatic effects in HCC cells (19, 20). In addition, many EMT-related lncRNAs signatures exhibit predictive potency for the survival of patients, which have been reported in bladder and kidney cancer, Colorectal Cancer, Melanoma, glioblastoma (21–25). However, the potential value of EMT-related lncRNAs as prognostic indicators is poorly explored in the HCC.

**FIGURE 1:**
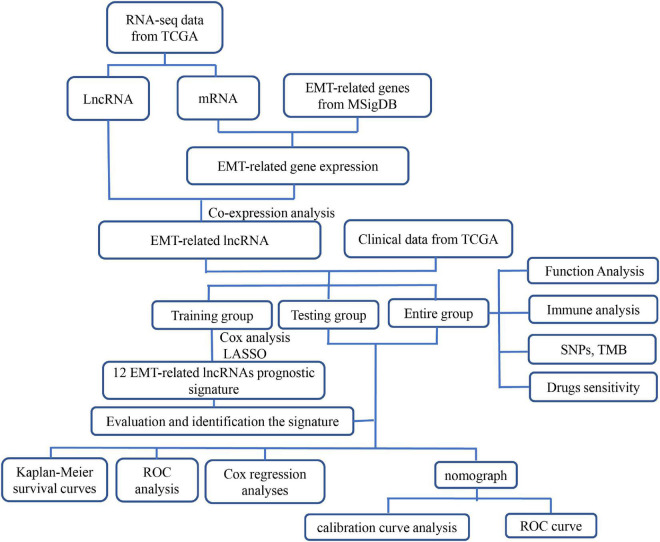
Flowchart of this study. ETM, epithelial-mesenchymal transition; LncRNA, long non-coding RNA; LASSO, the least absolute shrinkage and selection operator; GSEA, gene set enrichment analysis; GO, Gene Ontology; TCGA, The Cancer Genome Atlas; ROC, receiver operating characteristic; SNPs, single nucleotide polymorphisms; TMB, tumor mutation burden.

Therefore, the objective of this study was to construct and validate an EMT-related lncRNAs signature to determine the prognosis and drugs response of patients with HCC, we first constructed and identified an EMT-related lncRNAs prognostic model based on the data extracted from The Cancer Genome Atlas (TCGA) and then explored the association of the risk model with biological functions, immune cells infiltration, somatic mutations, and sensitivity of clinical drugs, in the patients with HCC. The flowchart of this study was shown in Figure 1.

## Materials and Methods

### Data Acquisition and Processing

The RNA sequencing (RNA-Seq) data in FPKM (fragments per kilobase of transcript per million mapped reads) format of HCC project (including 374 tumor and 50 normal samples), single nucleotide polymorphism (SNP) data (*n* = 362), and the corresponding clinical data (including survival status, survival time, age, gender, histological grade, and TNM stage) were downloaded from the TCGA database.^1^ The genes were annotated and sorted as 19,659 protein-coding genes and 14,142 lncRNAs according to the ENSEMBL database.^2^ The exclusion criteria of this study were set as follows: (1) Samples without intact genomics for analysis; (2) The genes with an average value <1; (3) The patients whose clinical follow-up time less than 30 days. The remaining data of 343 HCC samples with 710 lncRNAs and 11,282 protein-coding genes were included in our study. The patients were stochastically assigned into the training (*n* = 240) and testing group (*n* = 103) at the rate of 7:3 using “caret” package of R v.4.0.4 software^3^ (26, 27). Both training and testing sets conformed to the following requirements: (1) samples were randomly assigned to training and testing group; (2) patients in two groups had similar clinicopathological features. A prognostic EMT-related lncRNAs risk model was established based on the training cohort, the testing and entire cohorts were used to verify the predictive performance of this model.

### Tissue Sample Collection and Ethics Approval

We collected 30 paired HCC tissues and non-cancerous normal tissues following informed consent from patients who underwent tumor surgical resection at the Second Affiliated Hospital of Nanchang University from October 2020 and November 2021. This study met the standards set by terms of the Declaration of Helsinki and was approved by the Second Affiliated Hospital of Nanchang University Medical Research Ethics Committee. Tissue samples were maintained at –80°C until RNA extraction.

### Screening for the Epithelial–Mesenchymal Transition-Related LncRNAs in Hepatocellular Carcinoma

A total of 200 EMT-related genes were downloaded from MSigDB v.7.4 (hallmark_ epithelial_ mesenchymal_ transition M5930).^4^ Then, 159 EMT-related genes with average value >1 were extracted from LIHC project of TCGA database by utilizing the “limma” R package. Pearson correlation coefficient analysis was performed to identify the EMT-related lncRNAs according to the criteria: | correlation coefficient | > 0.3 and *p* < 0.001. A total of 449 EMT-related lncRNAs were screened for further analysis.

### Construction and Verification of an Epithelial–Mesenchymal Transition-Related LncRNAs Prognostic Signature for Hepatocellular Carcinoma Patients

To estimate the prognostic value of EMT-related lncRNAs, we applied multiple analyses to build the prognostic risk signature in the training group. First, we performed univariate Cox regression and Kaplan-Meier survival analysis to identify the survival associated lncRNAs, the significant lncRNAs were defined as *p* < 0.05. Then, we incorporated these lncRNAs into the least absolute shrinkage and selection operator (LASSO) and multivariate Cox regression to construct a prognostic signature for HCC patients. Eventually, 12 lncRNAs were enrolled into the risk model. According to the multivariate Cox regression coefficient and expression value of each lncRNA, the risk score of each patient was calculated using following equation:


$$Riskscore=\sum_{i=1}^{n}\left(coef_{i}\times \beta_{i}\right)$$

where coef represents the regression coefficient and βis the expressive value of each EMT related lncRNA. The HCC patients in the training cohort were divided into high- or low-risk groups based on the median risk score. Similarly, the patients in testing and entire cohorts were allocated to either high-risk or low-risk groups. Thereafter, we conducted the Kaplan-Meier survival analysis to compare the overall survival (OS) between the high- and low-risk groups. Time-dependent receiver operating characteristic (time-ROC) curves were sketched to evaluate the predictive accuracy of the risk signature for 1-, 3-, and 5-year survival via “survivalROC” R package. Meanwhile, the stratification analyses were performed in entire cohort to assess the prognostic value of the risk signature in different populations, which based on the following clinicopathological features: age (≤65 and >65 years), gender (Male and Female), grade (Grade 1–2 and Grade 3–4), and clinical stage (Stage I–II and Stage III–IV); *p* < 0.05 was considered to be statistically significant.

Thereafter, Cox proportional-hazards analyses were performed to determine the independent factors for the prognosis of HCC patients, including the following clinicopathological factors: age, gender, grade, clinical stage, and risk signature; *p* < 0.05 was considered to be statistically significant. A multi-indicator receiver operating characteristic (multi-ROC) curves were drawn and the area under the curve (AUC) were calculated to compare the predictive ability between the risk signature and other clinicopathological features.

Additionally, the relevancy between the 12 prognostic EMT-related lncRNAs and EMT-related protein-coding genes was analyzed by using above Pearson correlation coefficient analysis and presented with co-expression network. The Cytoscape 3.8.2 software^5^ and “ggalluvial” R package were employed to visualize the co-expression correlation.

### Construction and Validation of the Predictive Nomogram

Based on the risk score and conventional clinical characteristics (including age, gender, histological grade, TNM stage), we established a nomogram in training, testing, and entire cohort to quantitatively assess the 1- and 3-year survival probability of HCC patients using the “survival” and “rms” R packages. Moreover, we assessed the predictive performance of the nomogram with calibration curves and ROC curves using “rms” and “foreign” packages of R.

### Enrichment Analysis

The transcriptome data of the entire group were selected for gene set enrichment analysis (GSEA) using the GSEA v.4.1.0 software.^6^ Based on the risk signature, 343 samples were divided into high- and low-risk groups, the enriched pathways were identified by GSEA analysis and Gene Ontology (GO) analysis in the two risk groups. In GSEA analysis, the c2.cp.kegg.v7.4.symbols.gmt downloaded from MSigDB^7^ was used as the reference and a false discovery rate (FDR) of q-value <0.05 was used to identify the significantly enriched pathways. The significant cancer-related pathways were presented in the result. Thereafter, differential expressed genes (DEGs) were extracted from TCGA database depending on the risk grouping and the statistical significance was defined as FDR < 0.05 and | log2FC| ≥ 1. According to the DEGs, GO analysis was conducted to evaluate the related biological pathways in the two risk subgroups. Corrected *p*-values <0.05 were considered significant, and the top 10 most significant biological pathways were displayed with bar charts and bubble plots.

### Immune Cells Infiltration and Immune-Related Pathways

According to the EMT-related lncRNAs prognostic model, single-sample gene set enrichment analysis (ssGSEA) was conducted to explore the correlation between the risk signature and immune cells infiltrating and the immune-related pathways. First, 16 immune infiltration cells and 13 immune-related pathways scores were calculated in each LIHC sample based on ssGSEA method using “GSVA,” “limma,” and “GSEABase” R package. Then, we compared the difference of immune cells infiltration and immune-related pathways in different risk groups. The result was visualized with boxplot using the “ggpubr” R package, and *p* < 0.05 was considered as statistical significance.

### Exploring the Correlation of the Risk Signature With Single Nucleotide Polymorphisms

The format (MAF) files of HCC SNPs mutation dataset, possessed by the method of “varscan,” were obtained from TCGA database. The genes mutation status, variant classification, and the tumor mutation burden (TMB) of each patient were acquired using the Perl 5.30.0 software.^8^ Then, we integrated the SNPs data and risk grouping, and explored the correlation between SNPs and risk signature. The top 20 driver genes with highest mutational frequencies and its mutation types in the high-risk and low-risk groups were displayed with waterfall plots using “GenVisR” R package. Next, chi-square test was performed to compared the gene mutational frequency between two risk groups. Kaplan–Meier survival analysis was applied to evaluate the prognostic role of TP53 mutation status, TMB, as well as TMB combined with risk signature in patients with HCC via “survival” package of R. *p* < 0.05 indicated a statistical significance.

### The Role of the Risk Signature in Predicting Treatment Response to Chemotherapy, Targeted Therapy, and Immunotherapy

To investigate the role of EMT-related lncRNAs risk signature in predicting the sensitivity of chemotherapy and targeted therapy of HCC patients, we applied the pRRophetic algorithm to calculate the drugs half-maximal inhibitory concentration (IC50) based on the Cancer Cell Line Encyclopedia (CCLE) by using “pRRophetic” R package (28). According to the National Comprehensive Cancer Network (NCCN) guidelines, antitumor drugs such as doxorubicin, camptothecin, cisplatin, mitomycin C, gemcitabine, and sorafenib were selected for this analysis. The IC50 of these drugs were compared between the high- and low-risk groups with Wilcoxon rank-sum test, and *p*-value <0.05 was considered as significant difference.

It was reported that the immune checkpoint related genes can effectively reflect the response rate of immune checkpoint inhibitors (ICIs), and immunophenoscore (IPS) associated with the responds to ICIs (29, 30). Thus, we further investigated the association of risk signature with immune-related genes and IPS. We first carried out differential expression analysis of 46 immune checkpoint related genes in high- and low-risk groups and focused on the correlation of risk signature with six key genes: CTLA4, PD-1, IDO1,TIM-3 PD-L1, and PD-L2 (29). Then, IPS [four subtypes: IPS-CTLA4(+)/PD-1(+), IPS-CTLA4(+)/PD-1(–),IPS-CTLA4(–)/PD-1(+), IPS-CTLA4(–)/PD-1(–)] were compared in high- and low-risk groups with Wilcoxon rank-sum test, which were obtained from the Cancer Immunome Atlas database.^9^ *p*-value <0.05 was deeded statistically significant.

### Cell Culture and Real-Time Reverse Transcription-Quantitative Polymerase Chain Reaction

Human HCC cell lines (HCC-LM3, SMMC-7721) were purchased from the Cell Bank of Typical Culture Preservation Committee of Chinese Academy of Sciences (Shanghai, China). The other three HCC cell lines (SMMC97-H, HepG2, Huh7) and one normal liver cell line (L-02) were purchased from Procell Life Science and Technology Co., Ltd. (Wuhan, China). Cells were cultured in high glucose Dulbecco’s modified Eagle medium (DMEM) with 10% FBS (Gibco, Grand Island, NY, United States), 100 μg/mL streptomycin and 100 U/mL penicillin sodium (Biotechnology, Beijing, China). The cells were incubated at 37°C with 5% CO^2^ in a humidified incubator.

Total RNA was isolated from HCC cells and tissue samples using Trizol Reagent (Invitrogen, Carlsbad, CA, United States) according to the product manual. Subsequently, the RNA was reversely transcribed to complementary DNA (cDNA) using the PrimeScript™ RT reagent kit with gDNA Eraser (RR047A, TaKaRa, China) according to the product instructions. The qPCR was conducted with TB Green^®^ Premix Ex Taq™ II (RR820A, TaKaRa, China) on a CFX96 Real-Time PCR Detection System. The reaction conditions were 94.0°C for 30 s, followed by 94.0°C for 4 s, 58.0°C for 15 s, and 72°C for 15 s, for a total of 40 cycles. Relative mRNA expression of HCC cells was calculated by the 2^–ΔΔCt^ method, while 2^–ΔCT^ was used to reckon the mRNA expression in HCC tissues, and the data was normalized by the endogenous control of Glyceraldehyde 3-phosphate dehydrogenase (GAPDH). qPCR assays were performed in triplicates. The lncRNA Primers were listed in Supplementary Table 1.

### Statistical Analysis

Statistical analyses were performed using the R software v.4.0.4,^10^ Perl 5.30.0 software (see text footnote 8) and GraphPad Prism 8.0 software.^11^ Chi-square test was used to analyze the baseline characteristics of patients with HCC. Student’s *t*-test or Wilcoxon rank-sum test was applied to compare differences between the two groups. LASSO and Cox regression analyses were used to build a prognostic model. Kaplan-Meier analysis with log-rank test was used to evaluate the survival differences of the two groups. The ROC curve was used to estimate the predictive ability of the risk models. The predictive values of the nomogram for 1- and 3-year OS were assessed using calibration and ROC curves. All statistical tests were two-sided and *p* < 0.05 was considered statistically significant.

## Results

### Identification of Epithelial–Mesenchymal Transition-Related LncRNAs in Hepatocellular Carcinoma

A total of 424 HCC samples with transcriptome data (374 tumor samples and 50 normal samples) and 377 HCC specimens containing clinical information were acquired from TCGA database. After removing the repeated samples, and patients with incomplete clinicopathological data and less than 30 days follow-up time, the remaining 343 patients with complete follow-up information and 319 patients with complete clinicopathological data were included in this study for subsequent analysis. Of the 343 samples, 240 patients were randomly assigned to the training set, 103 patients were distributed to testing set. The clinicopathological features of the HCC patients included in this study are shown in Table 1. Then, 200 EMT-related genes were downloaded from MSigDB. After excluding low expression genes, the left 159 EMT-related genes were identified in TCGA dataset (Supplementary Table 2). According to the Pearson correlation coefficient analysis, 449 EMT-related lncRNAs were screened based on the filtering criteria of correlation coefficient < 0.3 and *p* < 0.001.

**TABLE 1:** Characteristics of hepatocellular carcinoma patients from TCGA dataset.

Characteristics	Training group (*n* = 224)	Testing group (*n* = 95)	Entire group (*n* = 319)	*P*-value
**Age (year)**				
≤60	113	47	160	0.987
>60	111	48	159	
**Gender**				
Male	156	63	219	0.842
Female	58	32	100	
**Tumor grade**				
G1	31	13	44	0.456
G2	117	37	154	
G3	68	41	109	
G4	8	4	12	
**Clinical stage**				
I	118	42	160	0.66
II	48	28	76	
III	55	25	80	
IV	3	0	3	

### Establishment the Epithelial–Mesenchymal Transition-Related LncRNAs Prognostic Model in the Training Cohort

**FIGURE 2:**
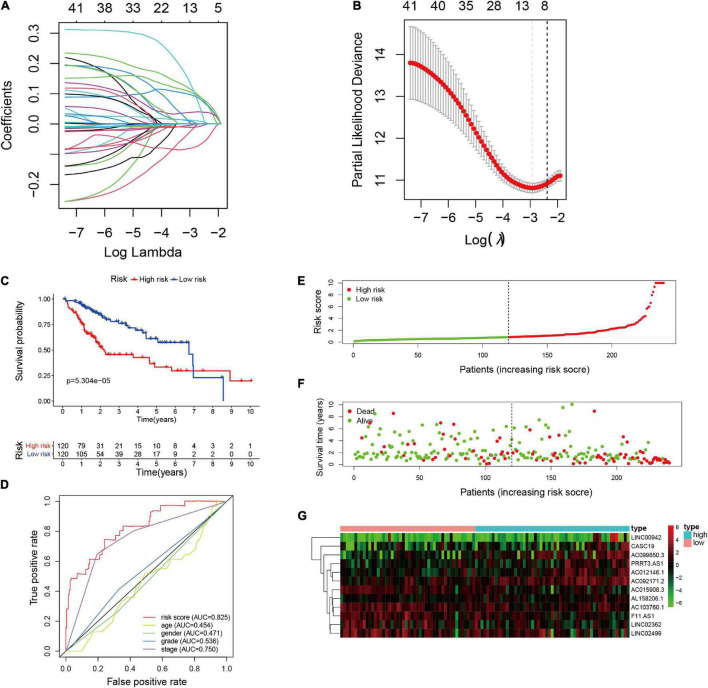
Construction of the EMT-related lncRNAs risk signature in training cohort. **(A)** LASSO coefficient profiles of the EMT-related lncRNAs. **(B)** Cross validation for tuning parameter selection in the proportional hazards model. **(C)** Kaplan-Meier survival analysis of the high- and low-risk groups. **(D)** The precision of the prognostic model was evaluated with Multi-indicator receiver operating characteristic (ROC) curves. **(E)** Risk score curves based on the risk score of each patient. **(F)** Scatter plots based on the survival status of each patient. **(G)** Heatmap displaying the expression levels of the 12 EMT-related lncRNAs in high- and low-risk groups.

We identified 449 EMT-related lncRNAs in above analysis, of which 41 were significantly correlated with the survival of HCC patients based on univariate Cox regression analysis and Kaplan-Meier analysis in the training set (Supplementary Table 3). Thereafter, LASSO-penalized Cox regression and multivariate Cox regression analysis were performed to identify the prognostic lncRNAs, the most relevant prognostic parameters were calculated and cross-validated to establish the best prognostic model in the training set (Figures 2A,B). Eventually, 12 lncRNAs were selected to construct a prognostic signature with multivariate Cox regression coefficients, including AC103760.1, AC015908.3, LINC02362, LINC02499, F11-AS1, LINC00942, PRRT3-AS1, AC012146.1, AC092171.2, AC099850.3, CASC19, and AL158206.1 (Table 2). Besides, we constructed co-expression network of the 12 EMT-related lncRNAs and EMT-related protein-coding genes by using Cytoscape to visually present the correlation between EMT-related genes and EMT-related lncRNAs (Supplementary Figure 1).

**TABLE 2:** Multivariate Cox regression analysis of EMT-related lncRNA.

LncRNA	Coefficient	HR	HR.95L	HR.95H	*p*-value
AC012146.1	0.033	1.034	0.898	1.191	0.645
CASC19	0.163	1.177	1.066	1.301	0.001
AC092171.2	0.122	1.13	1.024	1.246	0.015
PRRT3.AS1	0.03	1.031	0.95	1.118	0.465
AC103760.1	–0.124	0.883	0.771	1.011	0.072
LINC00942	0.02	1.02	0.996	1.045	0.097
LINC02362	–0.028	0.973	0.911	1.039	0.409
AC099850.3	0.142	1.153	1.055	1.26	0.002
F11.AS1	–0.009	0.991	0.885	1.111	0.879
AC015908.3	–0.113	0.893	0.703	1.134	0.354
AL158206.1	0.313	1.368	1.072	1.745	0.012
LINC02499	–0.02	0.979	0.927	1.035	0.457

*LncRNA, long non-coding RNAs; HR, hazard ratio.*

*HR.95L: low 95% confidence interval (CI) of HR.*

*HR.95H: high 95% confidence interval (CI) of HR.*

Based on the expression value of each lncRNA and corresponding regression coefficient, the individualized risk score was calculated as the following formula: Risk score = (–0.124 × AC103760.1 expression) + (–0.113 × AC015908.3 expression) + (–0.027 × LINC02362 expression) + (–0.021 × LINC02499 expression) + (–0.009 × F11.AS1 expression) + (0.02 × LINC00942 expression) + (0.03 × PRRT3.AS1 expression) + (0.033 × AC012146.1 expression) + (0.122 × AC092171.2 expression) + (0.142 × AC099850.3 expression) + (0.163 × CASC19 expression) + (0.313 × AL158206.1 expression). HCC patients in the training set were divided into low-risk subgroup (*n* = 120) and high-risk subgroup (*n* = 120) basing on the median risk score (median value = 0.8488). Kaplan-Meier survival curve indicated that the HCC patients in the high-risk group had worse OS than those in the low-risk group (*p* = 5.304e-06), as shown in Figure 2C. Time-ROC curve analysis was performed to evaluate the precision of the prognostic model, and the results demonstrated that the risk model provided a precise predictive effect for 1-, 3-, and 5-year OS, the AUC values were 0.828, 0.811, and 0.758, respectively (Supplementary Figure 2A). Risk score curves and scatter plots revealed that the higher the score, the higher the mortality rate observed in the patients (Figures 2E,F). The heatmap showed that seven lncRNAs (LINC00942, PRRT3-AS1, AC012146.1, AC092171.2, AC099850.3, CASC19, and AL158206.1) were upregulated in the high-risk group, while the other five lncRNAs (AC103760.1, AC015908.3, LINC02362, LINC02499, and F11-AS1) were upregulated in the low-risk group (Figure 2G).

Furthermore, univariate and multivariate cox proportional hazards regression analyses were performed to evaluate the independent prognostic factors for patients with HCC. The results showed that the risk signature [HR = 1.080, 95% CI (1.051–1.110), *p* < 0.001] and clinical stage [HR = 1.744, 95% CI (1.348–2.258), *p* < 0.001] were independent predictors for OS in patients with HCC (Table 3). Moreover, multi-indicator receiver operating characteristic (multi-ROC) curve analysis showed that the AUC value of the risk model was the highest at 0.825 in the training group (Figure 2D), which demonstrated that the EMT-related lncRNAs risk model provided a more precise prediction for OS than other clinical parameters in the patients with HCC.

**TABLE 3:** Univariate and multivariate Cox regression analyses of the risk score model and clinical factors.

Variables	Univariate analysis		Multivariate analysis	
	**HR (95% CI)**	***P*-value**	**HR (95% CI)**	***P*-value**
**Training cohort (*n* = 240)**				
Age (≤60/>60)	0.865 (0.549-1.364)	0.535	0.894 (0.565-1.414)	0.63
Gender (male/female)	1.078 (0.666-1.746)	0.757	1.03 (0.637-1.664)	0.905
Grade (G1+G2)/(G3+G4)	1.223 (0.766-1.957)	0.399	1.165 (0.717-1.894)	0.537
Stage (stage I+II/stage III+IV)	3.004 (1.898-4.755)	<0.001	2.541 (1.582-4.082)	**<0.001**
risk score (high/low)	2.798 (1.715-4.568)	<0.001	2.279 (1.369-3.792)	**0.001**
**Testing cohort (*n* = 103)**				
Age (≤/>60)	2.075 (0.963-4.469)	0.062	1.781 (0.798-3.975)	0.159
Gender (male/female)	1.789 (0.862-3.717)	0.119	1.208 (0.559-2.614)	0.631
Grade (G1+G2)/(G3+G4)	0.839 (0.395-1.785)	0.65	0.915 (0.407-2.055)	0.83
Stage (stage I+II/stage III+IV)	2.348 (1.124-4.905)	0.023	1.875 (0.877-4.007)	0.105
Risk score (high/low)	2.965 (1.311-6.704)	0.009	2.515 (1.049-6.028)	**0.039**
**Entire cohort (*n* = 343)**				
Age (≤60/>60)	1.197 (0.812-1.764)	0.364	1.171 (0.791-1.733)	0.4297
Gender (male/female)	1.239 (0.831-1.846)	0.293	1.067 (0.712-1.599)	0.753
Grade (G1+G2)/(G3+G4)	1.079 (0.726-1.607)	0.705	0.996 (0.66-1.504)	0.986
Stage (stage I+II/stage III+IV)	2.821 (1.913-4.159)	<0.001	2.442 (1.642-3.632)	**<0.001**
Risk score (high/low)	2.562 (1.695-3.871)	<0.001	2.206 (1.44-3.38)	**<0.001**

*HR, hazard ratio; 95%CI, 95% confidence interval (CI) of HR. The bold values represent p < 0.05 of multivariate Cox regression analysis.*

### Identification the Epithelial–Mesenchymal Transition-Related LncRNAs Risk Model in the Validation Cohorts

**FIGURE 3:**
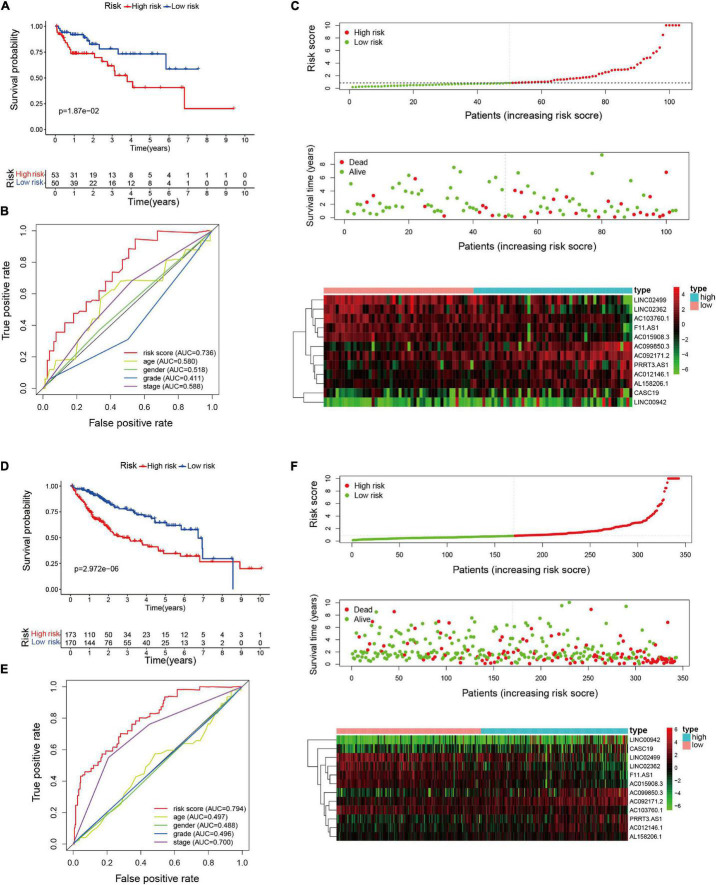
Validation of the EMT-related lncRNAs risk signature in the testing and entire cohorts. **(A)** Kaplan–Meier survival analysis of the high- and low-risk groups in testing cohort. **(B)** The precision of the prognostic model was assessed with multi-indicator receiver operating characteristic (ROC) curves in testing cohort. **(C)** Risk score curves, survival status, and heatmap of lncRNAs in the testing group. **(D)** Kaplan–Meier survival analysis of the high- and low-risk groups in entire cohort. **(E)** Multi-indicator receiver operating characteristic (ROC) curves in entire cohort. **(F)** Risk score curves, survival status, and heatmap of lncRNAs in the entire group.

To evaluate the applicability of the risk model, we conducted Kaplan-Meier analysis and Cox proportional hazards regression analysis in the testing and the entire cohorts. Firstly, we calculated the risk score of HCC patients in the validation cohorts according to the above-mentioned formula. The median risk score in the training cohort was applied to divide the patients (validation cohorts) into low-risk group or high-risk group. Kaplan–Meier analysis of the validation groups revealed that HCC patients with low risk had longer OS than those with high risk in testing set (*p* = 1.87e-02, Figure 3A) and entire set (*p* = 2.972e-06, Figure 3D). The AUC value of the risk model for predicting OS were 0.736 in the testing group and 0.794 in the entire group, indicating that it is more accurate than other clinical predictors in survival prediction (Figures 3B,E). The AUC values of the risk signature for 1-, 3-, and 5-year OS were 0.757, 0.675, and 0.682, respectively, in the testing group; and 0.809, 0.710, and 0.694, respectively, in the entire group (Supplementary Figures 2B,C). The results of the risk score curves, scatter plots, and heatmaps in the validation groups showed similar trends to those observed in the training group (Figures 3C,F). Cox proportional hazards regression analysis showed that EMT-related lncRNA risk model could act as an independent predictor for the survival of HCC patients in validation cohorts (Table 3).

**FIGURE 4:**
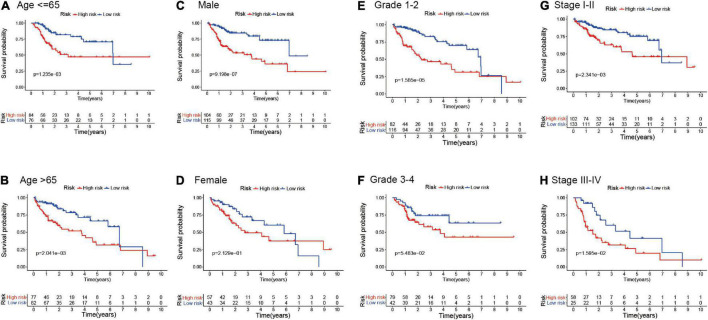
Kaplan–Meier survival analysis in different clinicopathological characteristic subgroups. **(A)** Age ≤ 60; **(B)** Age > 60; **(C)** Male; **(D)** Female; **(E)** Grade 1–2; **(F)** Grade 3–4; **(G)** Clinical stage I–II; **(H)** Clinical stage III–IV.

To further explore the prognostic value of the EMT-related lncRNAs risk signature in HCC patients, stratification analysis was performed in different subgroups. First, the patients in the entire cohort were divided into different subgroups based on age (≤65 and >65 years), gender (male and female), histological grade (grade 1–2 and grade 3–4), and clinical stage (stage I-II and stage III-IV). Then, Kaplan–Meier survival curve analysis was conducted in different subgroups. The results showed that patients with high risk in different age and clinical stage subgroups had worse survival than those with low risk, and the same results were observed in the male and Grade 1–2 subgroup. However, there was no significant difference in the female subgroup and patients with grade 3–4, which suggested that the prognostic signature was applicable in most subgroups of HCC (Figure 4).

### Differential Expression of the Epithelial–Mesenchymal Transition-Related LncRNAs in Hepatocellular Carcinoma Cells and Tissue Samples

**FIGURE 5:**
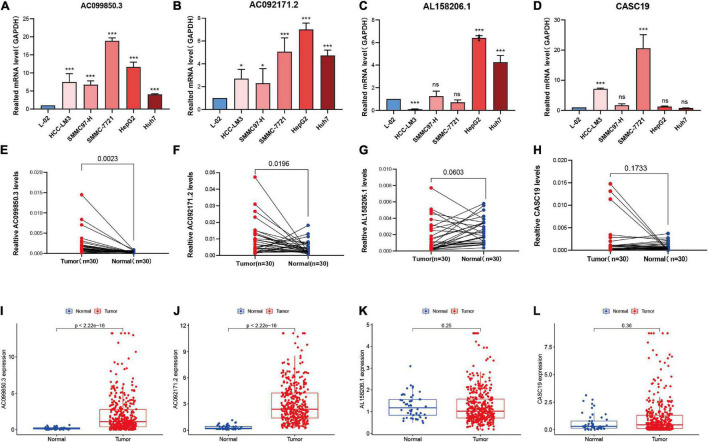
Expression level of lncRNAs in HCC cell lines, clinical tissue samples, and TCGA database. **(A–D)** The RT-qPCR analysis of the expression levels of AC099850.3, AC092171.2, AL158206.1, and CASC19 in five HCC cells and normal liver cell line (L-02). Error bars represent the SD of triplicate experiments. **(E–H)** The expression levels of AC099850.3, AC092171.2, AL158206.1, and CASC19 in 30 paired carcinoma tissues and adjacent tissues in HCC patients. **(I–L)** Expression analysis of AC099850.3, AC092171.2, AL158206.1, and CASC19 in HCC tissues and normal tissues derived from TCGA dataset. ns: not statistically significant, *p < 0.05 and ***p < 0.001.

The four lncRNAs (AC099850.3, AC092171.2, AL158206.1, and CASC19) with the greatest influence on the survival, which had confirmed in Cox proportional hazards regression analysis (Table 3), were selected for differential expression analysis in HCC cell lines and tissues samples. We first compared the four lncRNAs expression levels between normal liver cell line L-02 and five different HCC cell lines (HCC-LM3, SMMC97-H, SMMC-7721, HepG2, and Huh7) using real-time reverse transcription-quantitative polymerase chain reaction (RT-qPCR). The results showed that mRNA expression of AC099850.3 and AC092171.2 were higher in HCC cell lines compared with hepatic cell line (Figures 5A,B). Elevated mRNA expression of AL158206.1 and CASC19 was observed in part of human HCC cell lines (Figures 5C,D). Then, the differential expression analysis of the four lncRNAs was carried out in 30 paired HCC and para-cancer tissues by using RT-qPCR. The clinical information was displayed in Supplementary Table 4. The results of RT-qPCR revealed that AC099850.3 (*p* = 0.0023) and AC092171.2 (*p* = 0.0196) were significantly highly expressed in HCC tissues; whereas, no significant difference was observed in AL158206.1, and CASC19 between tumor tissues and para-carcinoma tissues (Figures 5E–H). These results were consistent with results from TCGA database, which conducted in 374 HCC tissues and 50 normal tissues with Wilcoxon rank-sum test (Figures 5I–L).

### A Nomogram Combining Risk Score With Other Clinical Features Predicting Overall Survival in Hepatocellular Carcinoma

**FIGURE 6:**
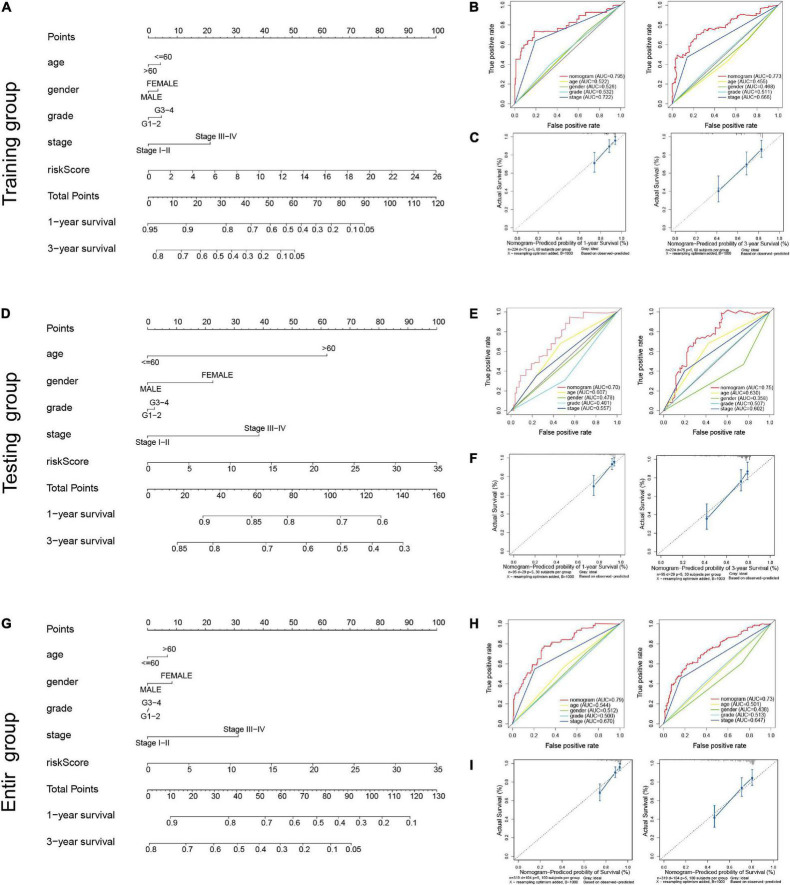
Construction of the predictive nomogram for HCC patients. **(A)** Nomogram predicting the survival probability of 1- and 3-year OS in training group; **(B)** multi-indicator receiver operating characteristic (ROC) curves of the nomogram for 1- and 3-year OS in training group; **(C)** calibration curve of the nomogram for predicting 1- and 3-year OS in training group; **(D)** nomogram predicting the survival probability of 1- and 3-year OS in testing group; **(E)** multi-indicator receiver operating characteristic (ROC) curves of the nomogram for 1- and 3-year OS in testing group; **(F)** calibration curve of the nomogram for predicting 1- and 3-year OS in testing group; **(G)** nomogram predicting the survival probability of 1- and 3-year OS in entire group; **(H)** multi-indicator receiver operating characteristic (ROC) curves of the nomogram for 1- and 3-year OS in entire group; **(I)** calibration curve of the nomogram for predicting 1- and 3-year OS in entire group.

We incorporated age, gender, grade, clinical stage, and risk score to establish a nomogram both in training cohort and validation cohorts to predict the 1- and 3-year OS for patients with HCC. The Comprehensive scores were obtained by combining each clinical factor; the higher the total score of patients, the worse the prognosis (Figures 6A,D,G). The predictive consistency and accuracy of the nomogram for 1- and 3-year OS were assessed using calibration curves and ROC curves. In training cohort, the calibration curves showed the nomogram model nearly in accordance with reality, and ROC curves demonstrated that the nomogram provided an accurate survival prediction with AUC = 0.795 for 1-year OS and 0.773 for 3-years OS as shown in Figures 6B–C. Similar results were showed in testing cohort (Figures 6E,F) and entire cohort (Figures 6H,I). These results demonstrate that the nomogram exhibited preferable clinical practicality in predicting the prognosis of HCC patients.

### Comparison of the Risk Signature and Other Epithelial–Mesenchymal Transition-Related Models in Hepatocellular Carcinoma

Next, we compared the predictive performance of the risk signature built in this study with other five EMT-related prognostic models for HCC, including the EMT-related five-lncRNA signature (31), EMT-related teen-gene signature (32), EMT-related five-gene signature (33), the EMT-related six-gene signature (34). We mainly compared the AUCs of ROC curves to evaluate the accuracy of these risk model. As shown in Table 4, the results indicated the risk signature built in our study exhibited a higher predictive efficacy with AUC values greater 0.7 in training group when compared with the previous EMT-related lncRNA models. Furthermore, an independent validation set was used to verify the predictive ability of the risk model, and a similar predictive performance was observed in test cohort, indicating this risk signature has good practicability and generalizability.

**TABLE 4:** Comparison of the risk model and other models.

Study	Signature	AUCs in the training set	AUCs in the validation set	AUCs of the nomogram
Our study	12 EMT-related lncRNAs	TCGA (*N* = 240) 0.828 for 1-year 0.811 for 3-year 0.758 for 5-year	TCGA (*N* = 103) 0.757 for 1-year 0.675 for 3-year 0.682 for 5-year	0.795 for 1-year 0.773 for 3-year
Xu et al. (31)	5 EMT-related lncRNAs	TCGA (*N* = 370) 0.754 for 1-year 0.704 for 3-year 0.662 for 5-year	No validation set	No AUCs of the nomogram
Wang et al. (32)	10 EMT-related genes	TCGA (*N* = 200) 0.858 for 1-year 0.846 for 3-year 0.824 for 5-year	TCGA (*N* = 146) 0.755 for 1-year 0.714 for 3-year 0.757 for 5-year	0.822 for 1-year 0.822 for 3-year 0.807 for 5-year
Zhu et al. (33)	5 EMT-related genes	TCGA (*N* = 319) 0.803 for 1-year 0.721 for 2-year 0.7 for 3-year	ICGC (*N* = 232) 0.739 for 1-year 0.704 for 2-year 0.754 for 3-year	0.762 for 1-year 0.724 for 3-year 0.676 for 5-year
Wu et al. (34)	6 EMT-related genes	TCGA (*N* = 365) 0.773 for 1-year 0.721 for 2-year 0.763 for 3-year	ICGC (*N* = 231) 0.699 for 1-year 0.757 for 2-year 0.760 for 3-year	No AUCs of the nomogram

*AUC, area under the curve; TCGA, The Cancer Genome Atlas; ICGC, International Cancer Genome Consortium.*

### Function Analysis of the Epithelial–Mesenchymal Transition-Related LncRNAs Prognostic Signature

**FIGURE 7:**
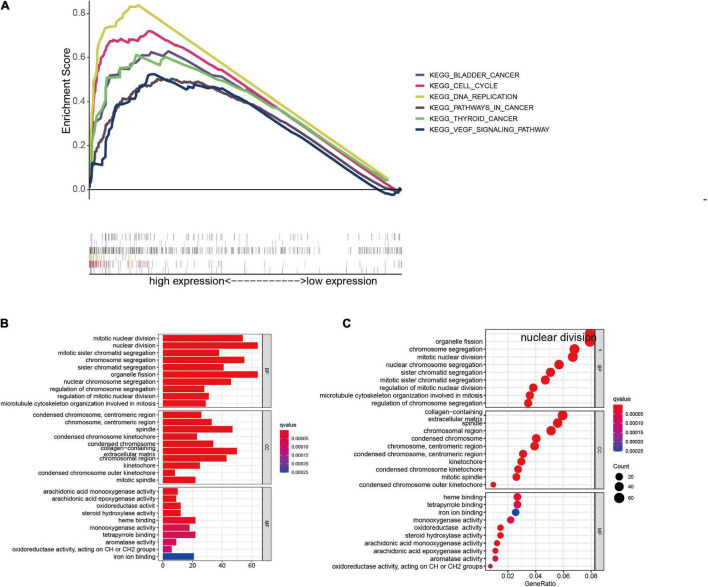
Functional analysis between the low- and high-risk group in the entire cohort. **(A)** GSEA analysis based on the risk grouping. **(B,C)** GO analysis between high- and low-risk groups. GSEA, gene set enrichment analysis; GO, Gene Ontology.

Gene set enrichment analysis was conducted in the entire group to probe the potential biological mechanism of the EMT-related lncRNAs signature. The results suggested tumor-related pathways, including bladder cancer, thyroid cancer, pathways in cancer, VEGF signaling pathway, cell cycle, and DNA replication were highly enriched in the high-risk group with FDR (*q*-value) < 0.05, as shown in Figure 7A.

To further determine the biofunctions associated with the risk signature, differentially expressed genes were extracted from the TCGA database based on risk grouping. A total of 969 DEGs (upregulated 832 genes, downregulated 146 genes, fold change > 1, *p* < 0.05) were identified in the high-risk group compared to the low-risk group. We then selected the DEGs for GO enrichment analysis, and the results showed that these DEGs were mainly enriched in terms associated with cell division process (Figures 7B,C), suggested that the proliferation ability of HCC cells in the high-risk group was notably more rapid than that in the low-risk group.

### The Relationship Between the Epithelial–Mesenchymal Transition-Related LncRNAs Prognostic Model and Tumor Immune Features

**FIGURE 8:**
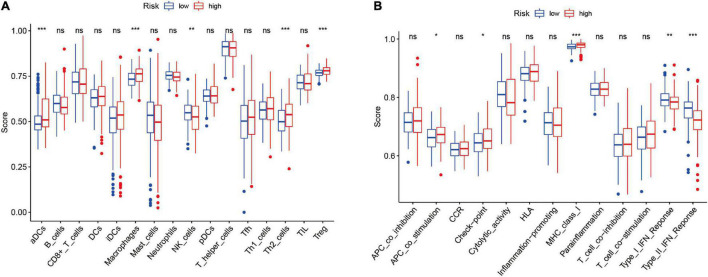
The relationship between the risk score, immune infiltration cells, and immune functions based on ssGSEA. **(A)** Comparison of the infiltration levels of 16 immune cells between the low- and high-risk groups. **(B)** Comparison of the 13 immune functions between the low- and high-risk groups. ns: not statistically significant, *p < 0.05; **p < 0.01; ***p < 0.001. ssGSEA: single-sample gene set enrichment analysis.

To further investigate the potential correlation between the risk signature and the tumor immunity in HCC, we employed ssGSEA analysis to compare the difference of 16 immune infiltrating cells and 13 immune function-related pathways between high- and low-risk groups in entire cohort. The results revealed that the infiltration level of immune cells, including aDCs, macrophages, Th2 cells, and Treg cells, were significantly upregulated in the high-risk groups, but natural killer (NK) cells were upregulated in the low-risk group (*P* < 0.05; Figure 8A). And we found antigen-presenting cell (APC) co-stimulation, checkpoint, and major histocompatibility complex-I (MHC-I) were significantly activated in the high-risk group; nevertheless, the pathways of INF-I and INF-II responses were evidently activated in the low-risk group (*P* < 0.05) (Figure 8B). These findings suggested that the risk signature could influence the tumor immune statues in patients with HCC.

### The Association of Risk Model With Single Nucleotide Polymorphisms

**FIGURE 9:**
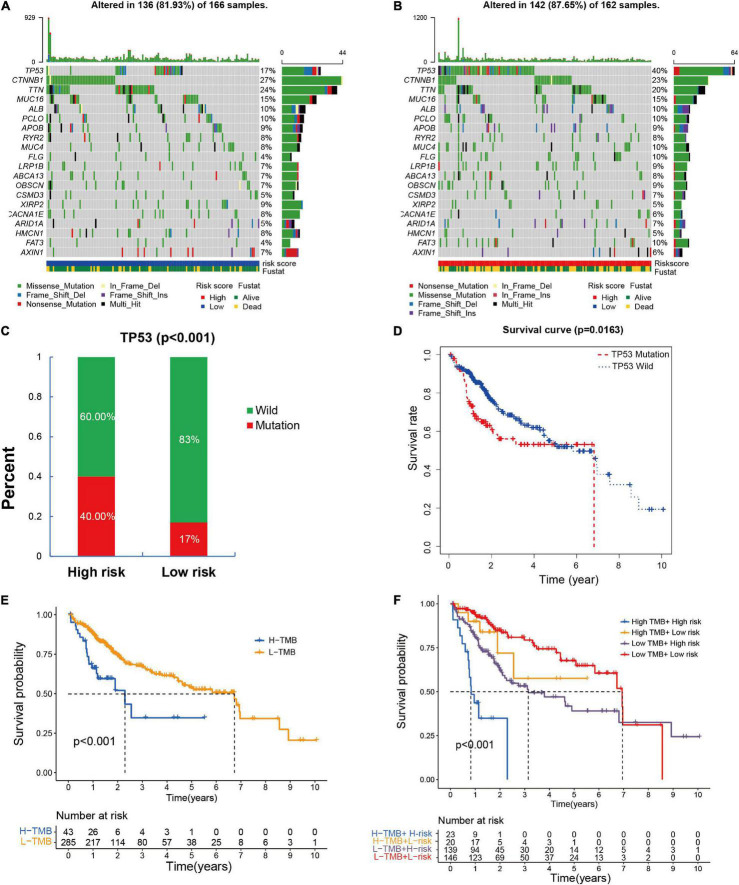
The association between risk signature and single-nucleotide variant in HCC. **(A,B)** The top 20 genes mutations in high- and low-risk groups based on the TCGA entire cohort; **(C)** the occurrence probability of TP53 mutations in high- and low-risk groups. **(D)** Kaplan–Meier survival analysis according to the mutation state of TP53. **(E,F)** Kaplan–Meier survival analysis based on the TMB and risk signature.

It has been confirmed that SNPs contributes to tumorigenesis and tumor progression. Tumor mutation burden (TMB) is defined as the total number of somatic mutations detected per million bases, including gene coding errors, base substitution, gene insertion or deletion errors. TMB has been reported to be used as a valid prognostic biomarkers and immune therapy response indicators in many kinds of tumor (35). Thus, we explored the connection among the SNPs, TMB, and the EMT-related lncRNAs signature. The top 20 mutation genes in high- and low-risk groups were shown in Figures 9A,B, the cumulative incidence plots showed that somatic mutations occurred more frequently in the high-risk group compared to the low-risk group (87.65 vs. 81.93%), and the prevalence of TP53 mutations was significantly different between high- and low-risk groups (*p* < 0.001) (Figure 9C). A significant association was observed between TP53 mutational status and prognosis from Kaplan–Meier analysis (*p* = 0.0163), indicating that the HCC patients with TP53 mutation had worse prognosis than those without TP53 mutation (Figure 9D). Meanwhile, we found the high TMB was also associated with a worse prognosis (*p* < 0.001) (Figure 9E). The patients with the low TMB and low-risk had the best overall survival compared with other groups, while the high TMB and high-risk group had the worst prognosis (Figure 9F). Collectively, it can be concluded that the risk signature may relevant to the somatic mutations, thus contribute to tumor progression.

### Differential Response to Chemotherapy, Targeted Therapy, and Immunotherapy Based on Risk Signature

**FIGURE 10:**
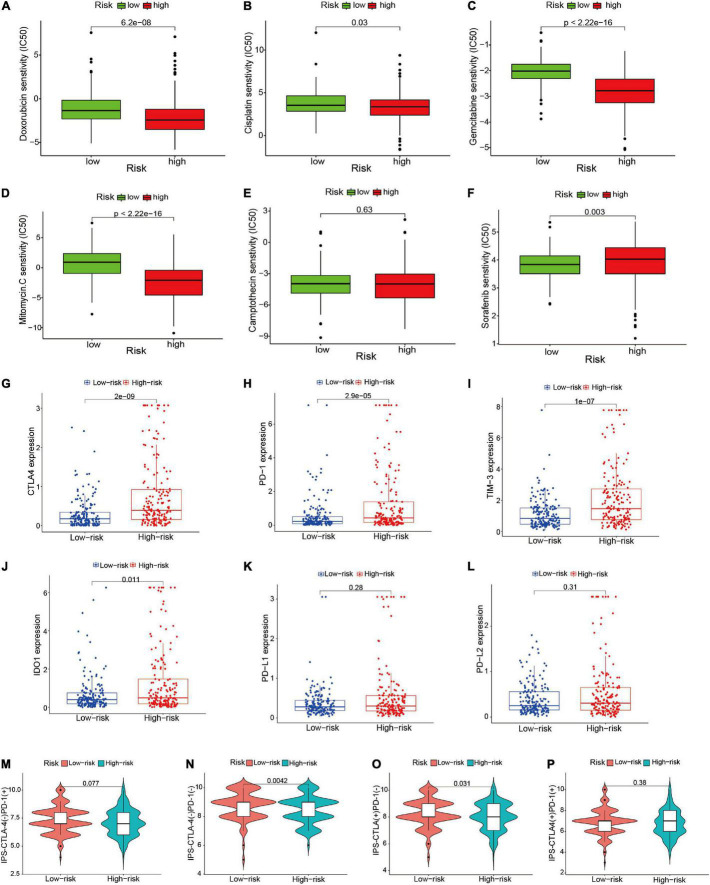
Comparison of treatment drugs sensitivity between high- and low-risk groups. **(A–F)** IC50 of doxorubicin, cisplatin, gemcitabine, mitomycin C, camptothecin, and sorafenib in high- and low-risk groups. **(G–L)** The difference of the expression level of CTLA4, PD-1, TIM-3, IDO-1, PD-L1, and PD-L2 between high- and low-risk groups. **(M–P)** Comparison of the treatment response of ICIs in two risk score groups. ICIs, immune checkpoint inhibitors; IC50, half-maximal inhibitory concentration.

To explore the different response to drugs treatment between high- and low-risk groups, we applied the pRRophetic algorithm to estimate the IC50 of chemotherapeutic agents and targeted drugs, and compared the IC50 of different drugs in two risk groups with Wilcoxon rank-sum test. Doxorubicin, camptothecin, mitomycin C, cisplatin, gemcitabine, and sorafenib were included in this analysis. As shown in Figures 10A–F, we found higher IC50 of Doxorubicin, camptothecin, mitomycin C, cisplatin, gemcitabine was presented in low-risk group, whereas low-risk group was associated with a higher sensitivity to sorafenib, and no significant differences was observed in camptothecin. These results might guide individualized chemotherapy or targeted therapy for different risk populations.

Subsequently, we investigated the potential roles of the risk signature in predicting ICIs response in HCC patients. First, we compared the expression level of 46 immune related genes between high- and low-risk groups. We found 34 of the 46 immune genes significantly differentially expressed in different risk subgroups (*P* < 0.05) (Supplementary Figure 3). Among the six key genes (CTLA4, PD-1, IDO1, TIM-3, PD-L1, and PD-L2), the expression levels of CTLA4, PD-1, IDO1, and TIM-3 were significantly increased in the high-risk group, whereas there was no distinct difference in the expression of PD-L1 and PD-L2 between high- and low-risk groups (Figures 10G–L). This result might provide better guidance for drugs selection of ICIs in HCC patients. Moreover, we further explored the relationship between the risk signature and the IPS in HCC samples. The results indicated that patients in the low-risk group showed higher IPS in two subtypes [IPS-CTLA4(–)/PD-1(–), IPS-CTLA4(+)/PD-1(–)]; however, there no significant association between risk signature and the other two IPS subtypes [IPS-CTLA4(+)/PD-1(+), IPS-CTLA4(–)/PD-1(+)] (Figures 10M–P). The results indicated that the risk signature might contribute to the evaluation of response to ICBs in HCC patients.

## Discussion

Hepatocellular carcinoma is one of the most common malignant tumors worldwide, characterized by high recurrence rates, high mortality rates, and poor prognosis in patients (36). Growing evidence suggests that EMT plays a crucial role in the progression, invasion, and metastasis of various tumors (37). Thus, we focused on the EMT as the cut-in point to discover novel prognosis markers for HCC. In this study, an EMT-related 12-lncRNAs risk model was constructed and validated for HCC patients based on TCGA dataset, we found that the risk model could independently and accurately predict the OS of HCC patients in training cohort and testing cohort. In addition, we discovered that the 12-lncRNAs risk signature is connected to the immune infiltration level, key gene mutations, and drugs sensitivity in patients with HCC.

Previous researches have revealed that lncRNAs participate in the regulation of EMT and ultimately affect tumor progression and metastasis. Moreover, EMT-related lncRNAs could serve as novel and effective biomarkers and therapeutic targets for various tumors (38). However, the prognostic value of the EMT-related lncRNAs has rarely been investigated in HCC. Hence, in this study, we focused on constructing and validating a new EMT-related lncRNAs prognostic signature for HCC. Based on RNA-Seq and clinical information from the TCGA dataset, we screened out 12 EMT-related lncRNAs to construct a prognostic risk signature using Cox regression and LASSO regression in training cohort. Kaplan–Meier analysis indicated that the survival of HCC patients in the high-risk group was worse than those in the low-risk group, and the risk model achieved well prediction performance with AUC = 0.828 for 1-year OS, AUC = 0.811 for 3-year OS, and AUC = 0.758 for 5-years OS. The lncRNAs signature exhibited more sensitivity and specificity, and acted as independent indicators for HCC patients when compared with other clinical parameters. More importantly, the prognostic availability of the risk model was validated in the testing and entire groups. Besides, a nomogram, established basing on age, gender, grade, clinical stage, and risk score, provided better clinical practicality than the traditional tumor grade or clinical stage system. In comparison with the recently published studies that explored the EMT-related lncRNAs or other prognostic predictors in HCC (31), Our study demonstrates certain strengths. First, 343 HCC samples obtained from TCGA database were randomly assigned to training and testing group, the 12 EMT-related lncRNAs signature was built based on training cohort, and we tested the performance of the model with independent testing cohort. Next, the risk signature showed a high predictive value with AUC values between 0.7 and 0.9 both in training group and validation groups. Furthermore, the differential expression of some lncRNAs in this risk model were firstly identified in HCC cell lines and HCC tissues. In addition, we comprehensively explored the correlation of risk signature with immune cell infiltration feature, chemosensitivity, immunotherapy response, and somatic mutations status.

Some lncRANs in this risk model have been reported to be associated with the malignant phenotypes of HCC or other cancers. For instance, LINC02499 was significantly downregulated in HCC tissues compared to para-cancerous tissues, and its low expression was also remarkably correlated with poorer overall survival (39). lncRNA F11-AS1 was low expression in HCC tissues and attenuated tumor growth and metastasis via the F11-AS1/miR-3146/PTEN axis (40). In addition, the low expression of lncRNA F11-AS1 was correlated with poor prognosis in patients with HBV-related HCC via regulation of the miR-211-5p/NR1I3 pathway (41). LINC00942 acts as an oncogene in breast cancer that promotes cell proliferation and colony formation and inhibits cell apoptosis (42). The lncRNA CASC19 is markedly upregulated and is associated with tumor progression and poorer prognoses in various cancers, including colorectal cancer, advanced gastric cancer, non-small lung cancer cells, and pancreatic cancer (43–46). However, the other lncRNA in this risk model, including AC103760.1, AC015908.3, LINC02362, AC012146.1, AC092171.2, AC099850.3, and AL158206.1, have been rarely reported. In this study, we verified the expression of the four lncRNAs (AC099850.3, AC092171.2, AL158206.1, and CASC19) in HCC, which exhibited the greatest impact on the risk score and prognosis. We found that the mRNA expression levels of AC099850.3 and AC092171.2 was significantly higher in HCC cells and tissues, while the AL158206.1 and CASC19 is upregulated in part HCC cell lines, and no significant difference was observed in CASC19 and AL158206.1 expression between HCC tissues and para-cancerous tissues. Thus, the risk signature could discover novel biomarkers for further research.

Epithelial–mesenchymal transition promote tumor progression and metastasis not only through reprogramming of cancer cells but also through reshaping tumor microenvironment (TME) (47). Numerous studies have indicated that tumor cells undergoing EMT acquire the ability to secrete some of cytokines, chemokines and growth factors that could regulate immune infiltrating cells in TME, thus promoting cancer immune escape (10). In turn, the occurrence of EMT is closely associated with the TME. Zhang et al. (48) revealed that cancer-associated fibroblasts in TME are implicated in modulating enhancing EMT and suppressing the anti-tumor immunity of HCC cells, which ultimately benefit for HCC initiation with malignant phenotypes. The exosomes in the TME derived from distinct cell types mediate the drug resistance by regulating epithelial-mesenchymal transition (EMT) and immune response (49). Our previous study reported that some TME-related lncRNAs could be applied as an effective prognostic biomarker for HCC patients (50). In this study, we intensively explored the relationship of EMT-related lncRNAs signature with immune infiltrating cells and immune function-related pathways in TME. Thus, we provided insights into the EMT-mediated TME regulatory mechanisms by constructing a EMT-lncRNAs-TME interactive network. ssGSEA analysis revealed that immune cells, including aDCs, macrophages, Th2 cells, and Treg cells, were enriched in high-risk group, but NK cells were upregulated in the low-risk group. Besides, APC co-stimulation, check-point, and MHC-I were significantly upregulated in the high-risk group; while the INF-I and INF-II responses were upregulated in the low-risk group. It is generally known that Macrophages play a prominent role in tumorigenesis and cancer progression by presenting M1 and M2 polarization, and M2 polarization induces cellular proliferation, angiogenesis, and EMT in HCC (51). It has been reported that the infiltration of macrophages is proportional to Tregs and inversely proportional to CD8+ T cells in HCC, and the absence of NK cells may trend toward M2 macrophages and accelerate liver fibrogenesis (51). Given the above results, we speculated that the tumor immune response in the high-risk group was lower than that in low-risk group, which mainly owning to immunosuppressive cells, such as macrophages and Tregs. Conversely, the anti-tumor immune response pathway was enriched in the low-risk group, which may contribute to a favorable prognosis.

Increasing evidence has demonstrated that EMT is also involved in other malignant behaviors, such as irradiation resistance, chemoresistance, and targeted drug resistance in HCC (5, 52–55). According the risk signature constructed by EMT-related lncRNAs, the IC50 of common chemotherapeutic agents and targeted drugs were calculated to assess the drugs sensitivity in different risk groups. The results indicated doxorubicin, mitomycin C, cisplatin, gemcitabine seem to be more suitable for the patients in the high-risk group. However, sorafenib shows significant superiority in the low-risk group. These results may provide potential guidance for individualized chemotherapy and targeted therapy. In addition, the relationship among the immune checkpoint-related genes, IPS, and the TME-related lncRNA signature was further investigated, we found that 34 out of the 46 immune checkpoint -related genes were significantly different between the high- and low-risk groups, and the expression levels of CTLA4, PD-1, IDO, and TIM-3 were significantly upregulated in the high-risk group. Furthermore, HCC patients in the low-risk group exhibited higher IPS in two subtypes [IPS-CTLA4(–)/PD-1(–), IPS-CTLA4(+)/PD-1(–)] and tended to have a better response to ICIs. In short, our findings might guide personalized therapeutic strategies for patients with HCC.

There are a few limitations in this study. First, the 12 EMT-related lncRNAs risk signature was constructed and evaluated using limited data and clinical information from TCGA database, but without external validation from other database or clinical samples, which may limit the generalizability of our model. In addition, some of the lncRNAs reported in this study have not been previously studied; therefore, it is critical to verify the functional features and molecular mechanisms of the risk signature model. Next, we will continue to pay attention to the role of the signature in clinical practice and further conduct related experiments to explore the functions and mechanisms of the EMT-related lncRNAs. Besides, in recent years, single-cell multi-omics approaches and spatial transcriptomics are becoming available and wildly applied in discovering distinctive biomarkers and exploring tissue cytoarchitectures and functions (56, 57). Thus, we will further investigate the potential molecular and functional mechanisms of the 12 EMT-related lncRNA signatures in HCC basing on single-cell multi-omics and spatial transcriptomics.

In conclusion, our study established a novel EMT-related prognostic risk signature including 12 lncRNAs and constructed a nomogram to predict the overall survival of HCC patients, which may improve the prognostic predictive efficacy and guide the individualized treatment for the patients with HCC.

## Data Availability Statement

The datasets presented in this study can be found in online repositories. The names of the repository/repositories and accession numbers can be found in the article/Supplementary Material. Raw data are available from the corresponding author upon reasonable request.

## Ethics Statement

The studies involving human participants were reviewed and approved by the Second Affiliated Hospital of Nanchang University Medical Research Ethics Committee. The patients/participants provided their written informed consent to participate in this study.

## Author Contributions

SH and JW: conceptualization. SH: investigation. LZ, DL, and JZ: methodology. LZ and DL: supervision. LZ and JZ: visualization. SH: writing-original draft. JW: writing-review and editing. All authors have read and agreed to the published version of the manuscript.

## Conflict of Interest

The authors declare that the research was conducted in the absence of any commercial or financial relationships that could be construed as a potential conflict of interest.

## Publisher’s Note

All claims expressed in this article are solely those of the authors and do not necessarily represent those of their affiliated organizations, or those of the publisher, the editors and the reviewers. Any product that may be evaluated in this article, or claim that may be made by its manufacturer, is not guaranteed or endorsed by the publisher.

## Footnotes

1
https://cancergenome.nih.gov/

2
http://asia.ensembl.org/index.html

3
http://www.r-project.org/

4
http://www.broadinstitute.org/gsea/msigdb/index.jsp

5
http://www.cytoscape.org/

6
http://www.gsea-msigdb.org/gsea/index.jsp

7
http://www.gsea-msigdb.org/gsea/msigdb/collections.jsp

8
https://www.perl.org/

9
https://tcia.at/home

10
https://www.r-project.org/

11
http://www.graphpad-prism.cn

## Supplementary Material

Supplementary Figure 1 Co-expression network of the prognostic EMT-related lncRNAs and EMT-related genes.

Supplementary Figure 2 Time-dependent receiver operating characteristic (time-ROC) curve evaluated the precision of the prognostic model. **(A)** In training group; **(B)** in testing group; **(C)** in entire group.

Supplementary Figure 3 The correlation of TME-related lncRNA signature with the expression of 46 ICIs-related genes. ICIs, immune checkpoint inhibitors. ns, not statistically significant. **p* < 0.05; ^**^*p* < 0.01; ^***^*p* < 0.001.

## Abbreviations

APC: antigen-presenting cell
AUC: area under the curve
HCC: hepatocellular carcinoma
EMT: epithelial-mesenchymal transition
LncRNA: long non-coding RNA
FPKM: fragments per kilobase of transcript per million mapped reads
GO: Gene Ontology
GSEA: gene set enrichment analysis
HR: hazard ratio
IC 50: half-maximal inhibitory concentration
ICIs: immune checkpoint inhibitors
IPS: immunophenoscore
LASSO: the least absolute shrinkage and selection operator
MHC-I: major histocompatibility complex-I
multi-ROC: multi-indicator receiver operating characteristic
OS: overall survival
RT-qPCR: real-time reverse transcription-quantitative polymerase chain reaction
ROC: receiver operating characteristic
ssGSEA: Single-sample gene set enrichment analysis
SNPs: single nucleotide polymorphisms
TCGA: The Cancer Genome Atlas
TGF-β: Transforming growth factor-β
time-ROC: time-dependent receiver operating characteristic
TMB: tumor mutation burden.
