# Supplementary figures and images for: Identification of an Epithelial-Mesenchymal Transition-Related Long Non-coding RNA Prognostic Signature to Determine the Prognosis and Drug Treatment of Hepatocellular Carcinoma Patients
Source: Front Med (Lausanne). 2022 May 24;9:850343. doi: 10.3389/fmed.2022.850343 (PMC9170944; doi:10.3389/fmed.2022.850343)

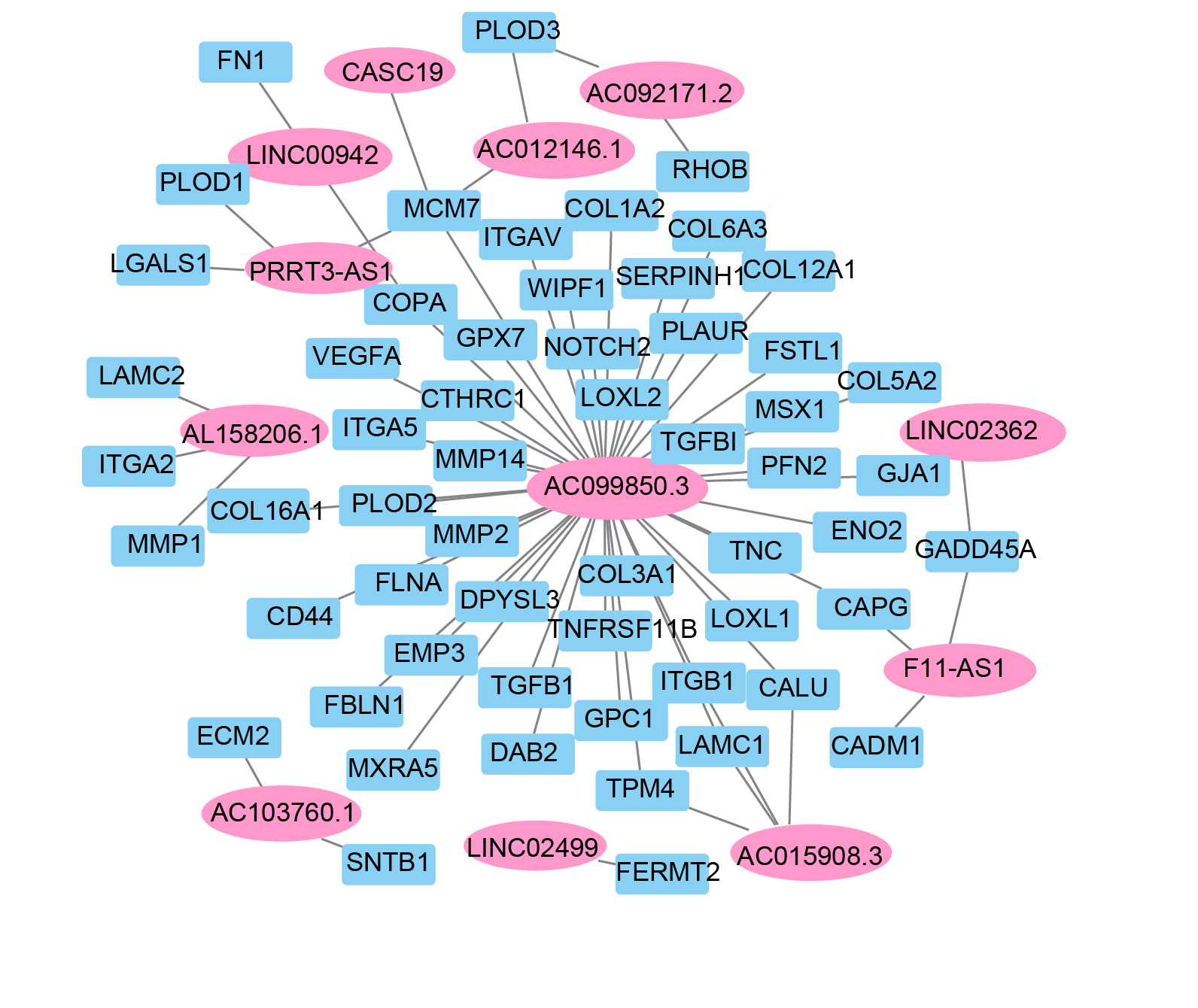

Supplement: Supplementary Figure 1 — Co-expression network of the prognostic EMT-related lncRNAs and EMT-related genes. [file Image_1.TIF]

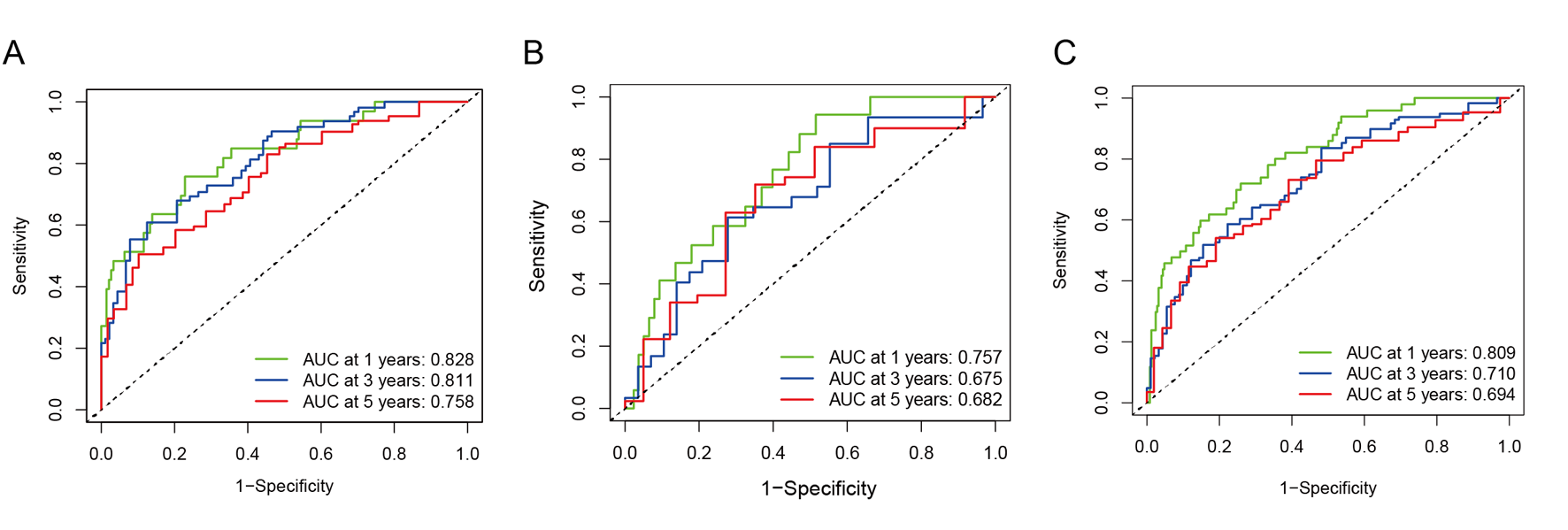

Supplement: Supplementary Figure 2 — Time-dependent receiver operating characteristic (time-ROC) curve evaluated the precision of the prognostic model. (A) In training group; (B) in testing group; (C) in entire group. [file Image_2.TIF]

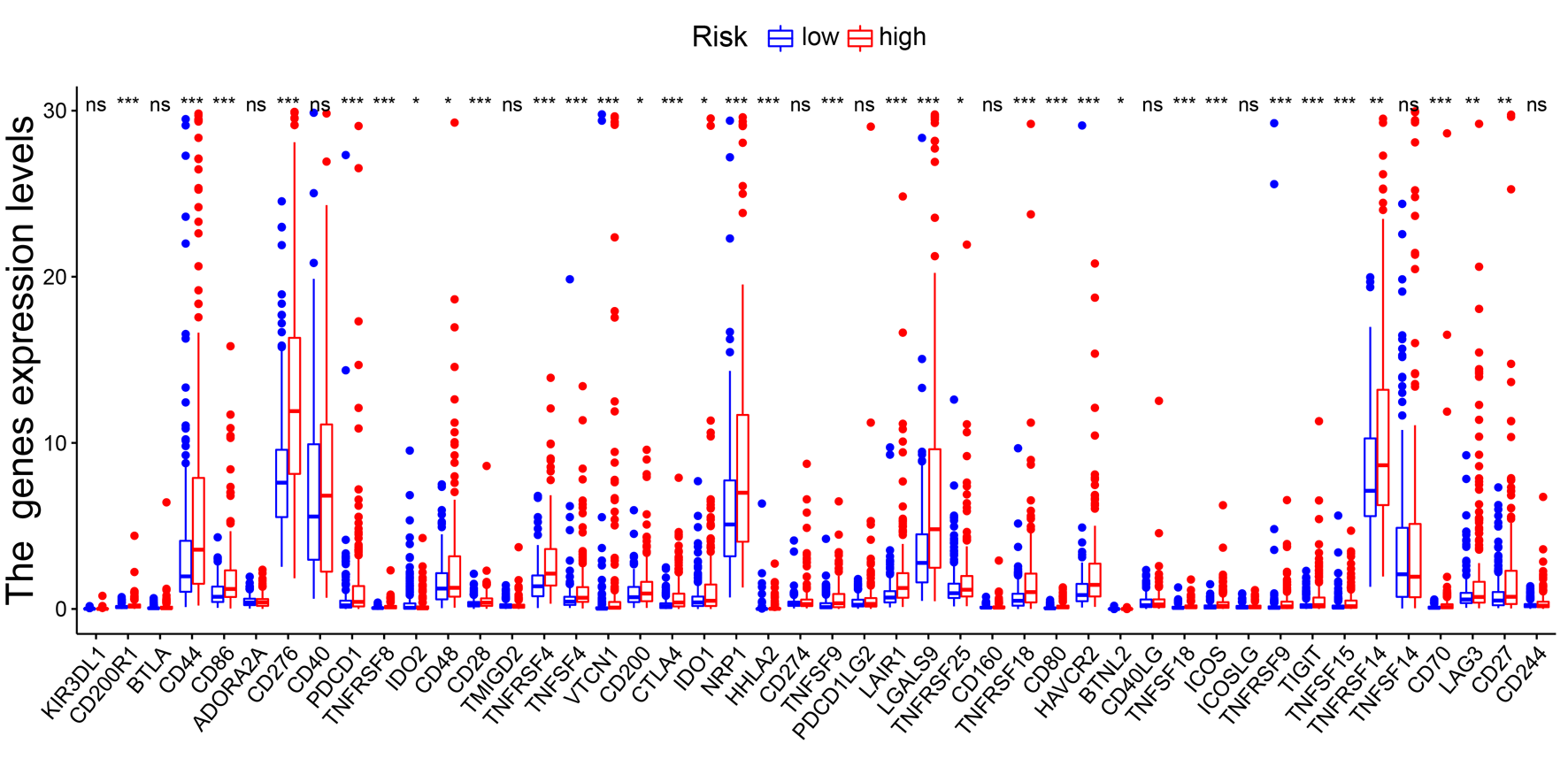

Supplement: Supplementary Figure 3 — The correlation of TME-related lncRNA signature with the expression of 46 ICIs-related genes. ICIs, immune checkpoint inhibitors. ns, not statistically significant. *p < 0.05; **p < 0.01; ***p < 0.001. [file Image_3.TIF]
