# Supplementary material for: Identification of an Epithelial-Mesenchymal Transition-Related Long Non-coding RNA Prognostic Signature to Determine the Prognosis and Drug Treatment of Hepatocellular Carcinoma Patients
Source: Front Med (Lausanne). 2022 May 24;9:850343. doi: 10.3389/fmed.2022.850343 (PMC9170944; doi:10.3389/fmed.2022.850343)
Supplement: Supplementary file 4 [file Table_1.DOCX]

Table S1. Primers for RT-qPCR analysis

| Name | Sequences (5'—3') |
| --- | --- |
| AC099850.3 | F: CGCTATGTTTCCCAGGCTGTATT; R: GGTCACTGTGAGACCTAGTTCCCT |
| AC092171.2 | F: CTGGGGTAGGAGTAGCCAAGTGT; R: AGGAGTTGGATGTTGCAGTGAGC |
| AL158206.1 | F: GGCAGTCAAGAATCTCCCTCCCT; R: GGGCAAATTCTGACCAGAACAAA |
| CASC19 | F: TCCTTGCTTTGTTCTGACTTTACG; R: TTCAAATCCAAGTGATAACCCATA |
| GAPDH | F: GGAGCGAGATCCCTCCAAAAT; R: GGCTGTTGTCATACTTCTCATGG |

F, forward; R, reverse.
