# Supplementary material for: Identification of an Epithelial-Mesenchymal Transition-Related Long Non-coding RNA Prognostic Signature to Determine the Prognosis and Drug Treatment of Hepatocellular Carcinoma Patients
Source: Front Med (Lausanne). 2022 May 24;9:850343. doi: 10.3389/fmed.2022.850343 (PMC9170944; doi:10.3389/fmed.2022.850343)
Supplement: Supplementary file 5 [file Table_2.DOCX]

Table S2: EMT-related genes obtained and screened from public database

| EMT-related genes downloaded from MSigDB | EMT-related genes screened from TCGA-LIHC dataset |
| --- | --- |
| ABI3BP | ACTA2 |
| ACTA2 | ANPEP |
| ADAM12 | APLP1 |
| ANPEP | AREG |
| APLP1 | BASP1 |
| AREG | BGN |
| BASP1 | BMP1 |
| BDNF | CADM1 |
| BGN | CALD1 |
| BMP1 | CALU |
| CADM1 | CAP2 |
| CALD1 | CAPG |
| CALU | CD44 |
| CAP2 | CD59 |
| CAPG | CDH2 |
| CD44 | COL12A1 |
| CD59 | COL16A1 |
| CDH11 | COL1A1 |
| CDH2 | COL1A2 |
| CDH6 | COL3A1 |
| COL11A1 | COL4A1 |
| COL12A1 | COL4A2 |
| COL16A1 | COL5A1 |
| COL1A1 | COL5A2 |
| COL1A2 | COL5A3 |
| COL3A1 | COL6A2 |
| COL4A1 | COL6A3 |
| COL4A2 | COL7A1 |
| COL5A1 | COMP |
| COL5A2 | COPA |
| COL5A3 | CTGF |
| COL6A2 | CTHRC1 |
| COL6A3 | CXCL1 |
| COL7A1 | CXCL12 |
| COL8A2 | CXCL6 |
| COMP | CYR61 |
| COPA | DAB2 |
| CRLF1 | DCN |
| CTGF | DKK1 |
| CTHRC1 | DPYSL3 |
| CXCL1 | DST |
| CXCL12 | ECM1 |
| CXCL6 | ECM2 |
| CYR61 | EDIL3 |
| DAB2 | EFEMP2 |
| DCN | ELN |
| DKK1 | EMP3 |
| DPYSL3 | ENO2 |
| DST | FAS |
| ECM1 | FBLN1 |
| ECM2 | FBLN2 |
| EDIL3 | FBLN5 |
| EFEMP2 | FBN1 |
| ELN | FERMT2 |
| EMP3 | FLNA |
| ENO2 | FMOD |
| FAP | FN1 |
| FAS | FSTL1 |
| FBLN1 | FSTL3 |
| FBLN2 | FUCA1 |
| FBLN5 | FZD8 |
| FBN1 | GADD45A |
| FBN2 | GADD45B |
| FERMT2 | GEM |
| FGF2 | GJA1 |
| FLNA | GLIPR1 |
| FMOD | GPC1 |
| FN1 | GPX7 |
| FOXC2 | HTRA1 |
| FSTL1 | ID2 |
| FSTL3 | IGFBP2 |
| FUCA1 | IGFBP3 |
| FZD8 | IGFBP4 |
| GADD45A | IL32 |
| GADD45B | INHBA |
| GAS1 | ITGA2 |
| GEM | ITGA5 |
| GJA1 | ITGAV |
| GLIPR1 | ITGB1 |
| GLT25D1 | ITGB5 |
| GPC1 | JUN |
| GPX7 | LAMA2 |
| GREM1 | LAMA3 |
| HTRA1 | LAMC1 |
| ID2 | LAMC2 |
| IGFBP2 | LGALS1 |
| IGFBP3 | LOX |
| IGFBP4 | LOXL1 |
| IL15 | LOXL2 |
| IL32 | LRP1 |
| IL6 | LUM |
| IL8 | MATN2 |
| INHBA | MATN3 |
| ITGA2 | MCM7 |
| ITGA5 | MEST |
| ITGAV | MGP |
| ITGB1 | MMP1 |
| ITGB3 | MMP14 |
| ITGB5 | MMP2 |
| JUN | MSX1 |
| LAMA1 | MXRA5 |
| LAMA2 | MYL9 |
| LAMA3 | MYLK |
| LAMC1 | NNMT |
| LAMC2 | NOTCH2 |
| LEPRE1 | NT5E |
| LGALS1 | PCOLCE |
| LOX | PCOLCE2 |
| LOXL1 | PDGFRB |
| LOXL2 | PFN2 |
| LRP1 | PLAUR |
| LRRC15 | PLOD1 |
| LUM | PLOD2 |
| MAGEE1 | PLOD3 |
| MATN2 | PMEPA1 |
| MATN3 | PMP22 |
| MCM7 | POSTN |
| MEST | PPIB |
| MFAP5 | PRSS2 |
| MGP | PTHLH |
| MMP1 | PVR |
| MMP14 | QSOX1 |
| MMP2 | RHOB |
| MMP3 | SAT1 |
| MSX1 | SDC1 |
| MXRA5 | SDC4 |
| MYL9 | SERPINE1 |
| MYLK | SERPINE2 |
| NID2 | SERPINH1 |
| NNMT | SFRP4 |
| NOTCH2 | SGCB |
| NT5E | SLC6A8 |
| NTM | SNAI2 |
| OXTR | SNTB1 |
| PCOLCE | SPARC |
| PCOLCE2 | SPP1 |
| PDGFRB | TAGLN |
| PDLIM4 | TGFB1 |
| PFN2 | TGFBI |
| PLAUR | TGFBR3 |
| PLOD1 | TGM2 |
| PLOD2 | THBS1 |
| PLOD3 | THBS2 |
| PMEPA1 | THY1 |
| PMP22 | TIMP1 |
| POSTN | TNC |
| PPIB | TNFAIP3 |
| PRRX1 | TNFRSF11B |
| PRSS2 | TNFRSF12A |
| PTHLH | TPM1 |
| PTX3 | TPM2 |
| PVR | TPM4 |
| QSOX1 | VCAM1 |
| RGS4 | VCAN |
| RHOB | VEGFA |
| SAT1 | VEGFC |
| SCG2 | VIM |
| SDC1 | WIPF1 |
| SDC4 | WNT5A |
| SERPINE1 |  |
| SERPINE2 |  |
| SERPINH1 |  |
| SFRP1 |  |
| SFRP4 |  |
| SGCB |  |
| SGCD |  |
| SGCG |  |
| SLC6A8 |  |
| SLIT2 |  |
| SLIT3 |  |
| SNAI2 |  |
| SNTB1 |  |
| SPARC |  |
| SPOCK1 |  |
| SPP1 |  |
| TAGLN |  |
| TFPI2 |  |
| TGFB1 |  |
| TGFBI |  |
| TGFBR3 |  |
| TGM2 |  |
| THBS1 |  |
| THBS2 |  |
| THY1 |  |
| TIMP1 |  |
| TIMP3 |  |
| TNC |  |
| TNFAIP3 |  |
| TNFRSF11B |  |
| TNFRSF12A |  |
| TPM1 |  |
| TPM2 |  |
| TPM4 |  |
| VCAM1 |  |
| VCAN |  |
| VEGFA |  |
| VEGFC |  |
| VIM |  |
| WIPF1 |  |
| WNT5A |  |
