# Supplementary material for: Identification of an Epithelial-Mesenchymal Transition-Related Long Non-coding RNA Prognostic Signature to Determine the Prognosis and Drug Treatment of Hepatocellular Carcinoma Patients
Source: Front Med (Lausanne). 2022 May 24;9:850343. doi: 10.3389/fmed.2022.850343 (PMC9170944; doi:10.3389/fmed.2022.850343)
Supplement: Supplementary file 6 [file Table_3.DOCX]

**Table S3. The 41 EMT-related lncRNAs significantly correlated with the survival of HCC patients**

| LncRAN | KM | B | SE | HR | HR.95L | HR.95H | p-value |
| --- | --- | --- | --- | --- | --- | --- | --- |
| NRAV | 0.000121 | 0.215683 | 0.056675 | 1.240709 | 1.110269 | 1.386472 | 0.000141 |
| ZFPM2-AS1 | 0.018612 | 0.06739 | 0.025499 | 1.069712 | 1.017566 | 1.124532 | 0.008221 |
| SNHG20 | 0.049071 | 0.248721 | 0.122301 | 1.282384 | 1.009056 | 1.629748 | 0.041984 |
| AC012146.1 | 0.025409 | 0.156785 | 0.055341 | 1.169744 | 1.049506 | 1.303758 | 0.00461 |
| TMEM220-AS1 | 0.004407 | -0.31239 | 0.094603 | 0.731694 | 0.607859 | 0.880757 | 0.00096 |
| SNHG16 | 0.029399 | 0.110652 | 0.054967 | 1.117006 | 1.002924 | 1.244066 | 0.044106 |
| CYTOR | 0.009916 | 0.061077 | 0.017409 | 1.062981 | 1.027322 | 1.099878 | 0.000451 |
| WAC-AS1 | 0.026997 | 0.092209 | 0.025913 | 1.096594 | 1.04229 | 1.153727 | 0.000373 |
| CASC19 | 0.041535 | 0.217209 | 0.047032 | 1.242604 | 1.13318 | 1.362594 | 3.87E-06 |
| AC092171.2 | 0.003849 | 0.183872 | 0.042764 | 1.201862 | 1.105233 | 1.306939 | 1.71E-05 |
| MIR210HG | 0.045375 | 0.110606 | 0.053323 | 1.116955 | 1.006114 | 1.240006 | 0.038053 |
| PRRT3-AS1 | 0.002382 | 0.133617 | 0.032171 | 1.142954 | 1.073111 | 1.217344 | 3.28E-05 |
| AC016747.1 | 0.014081 | 0.191948 | 0.063232 | 1.211608 | 1.070383 | 1.371466 | 0.0024 |
| AC103760.1 | 0.002707 | -0.14526 | 0.068159 | 0.864796 | 0.756654 | 0.988395 | 0.033071 |
| LINC02037 | 0.002934 | -0.13805 | 0.068802 | 0.871055 | 0.761169 | 0.996804 | 0.044804 |
| BX537318.1 | 0.023316 | 0.234862 | 0.097927 | 1.264734 | 1.043864 | 1.532338 | 0.016469 |
| LINC00942 | 0.019909 | 0.039038 | 0.00892 | 1.03981 | 1.021788 | 1.05815 | 1.21E-05 |
| DANCR | 0.012281 | 0.026629 | 0.008852 | 1.026987 | 1.009322 | 1.044961 | 0.002629 |
| LINC01138 | 0.002584 | 0.335931 | 0.113212 | 1.399242 | 1.120795 | 1.746865 | 0.003005 |
| BACE1-AS | 0.00972 | 0.257866 | 0.08093 | 1.294165 | 1.104338 | 1.516622 | 0.001441 |
| AC020915.3 | 0.016467 | 0.323503 | 0.117404 | 1.38196 | 1.097895 | 1.739522 | 0.005861 |
| SNHG3 | 0.002006 | 0.071229 | 0.018234 | 1.073827 | 1.036128 | 1.112898 | 9.37E-05 |
| AC009005.1 | 0.018975 | 0.156195 | 0.056481 | 1.169054 | 1.046544 | 1.305905 | 0.005685 |
| LINC02362 | 0.007346 | -0.07835 | 0.036646 | 0.924644 | 0.86056 | 0.9935 | 0.032524 |
| SNHG7 | 0.01739 | 0.038181 | 0.016974 | 1.03892 | 1.004924 | 1.074065 | 0.024491 |
| AC026401.3 | 0.022851 | 0.088374 | 0.027725 | 1.092397 | 1.034619 | 1.153401 | 0.001435 |
| AC099850.3 | 0.008972 | 0.188383 | 0.039572 | 1.207295 | 1.117196 | 1.304661 | 1.93E-06 |
| AC083799.1 | 0.03801 | 0.09689 | 0.038534 | 1.101739 | 1.021594 | 1.188172 | 0.011924 |
| AL049840.4 | 0.019244 | 0.080588 | 0.029941 | 1.083924 | 1.022146 | 1.149436 | 0.007112 |
| F11-AS1 | 0.022179 | -0.19582 | 0.071955 | 0.822159 | 0.714016 | 0.946682 | 0.0065 |
| SNHG12 | 0.031521 | 0.124075 | 0.061239 | 1.132101 | 1.004057 | 1.276473 | 0.042757 |
| AC015908.3 | 0.001372 | -0.39326 | 0.110638 | 0.674853 | 0.543293 | 0.838271 | 0.000379 |
| AC115619.1 | 0.006708 | -0.01609 | 0.006454 | 0.984043 | 0.971675 | 0.996569 | 0.012687 |
| AC010969.2 | 0.035915 | 0.29076 | 0.129824 | 1.337443 | 1.036976 | 1.724972 | 0.025114 |
| AL158206.1 | 0.025436 | 0.215189 | 0.108255 | 1.240097 | 1.003017 | 1.533214 | 0.046835 |
| AC009779.2 | 0.004318 | 0.125415 | 0.050719 | 1.133618 | 1.026347 | 1.252101 | 0.013409 |
| AL365203.2 | 0.005213 | 0.112562 | 0.037829 | 1.119141 | 1.039167 | 1.205271 | 0.002924 |
| LINC02499 | 0.013264 | -0.06393 | 0.028201 | 0.938069 | 0.887626 | 0.991377 | 0.023387 |
| OTUD6B-AS1 | 0.013129 | 0.149999 | 0.057154 | 1.161833 | 1.038709 | 1.299551 | 0.008679 |
| ZFAS1 | 0.013938 | 0.012732 | 0.005921 | 1.012814 | 1.001129 | 1.024635 | 0.031512 |
| LINC00261 | 0.00222 | -0.02255 | 0.008336 | 0.977703 | 0.96186 | 0.993808 | 0.006827 |

LncRNA: long noncoding RNAs; HR: hazard ratio;

KM: *p* value of Kaplan-Meier analysis

HR.95L: low 95% confidence interval (CI) of HR;

HR.95H: high 95% confidence interval (CI) of HR;
