# Supplementary material for: Identification of an Epithelial-Mesenchymal Transition-Related Long Non-coding RNA Prognostic Signature to Determine the Prognosis and Drug Treatment of Hepatocellular Carcinoma Patients
Source: Front Med (Lausanne). 2022 May 24;9:850343. doi: 10.3389/fmed.2022.850343 (PMC9170944; doi:10.3389/fmed.2022.850343)
Supplement: Supplementary file 7 [file Table_4.DOCX]

**Table S4. Clinical and pathological features of HCC patients**

| **Characteristics** | **Number of cases (%)** |
| --- | --- |
| **Age** |  |
| ≤60 | 19(63.3) |
| ＞60 | 11(36.7) |
| **Gender** |  |
| Male | 27(90) |
| Female | 3(10) |
| **HBsAg** |  |
| Negative | 7(23.3) |
| Positive | 23(76.7) |
| **Child-Pugh classification** | |
| A | 15(50) |
| B | 15(50) |
| **AFP** |  |
| ≤400 ng/ml | 18(50) |
| >400 ng/ml | 12(40) |
| **Liver cirrhosis** | |
| Absent | 7(23.3) |
| Present | 23(76.7) |
| **Tumor number** | |
| Single | 22(73.3) |
| Multiple | 8(26.7) |
| **Lymph nodes metastasis** | |
| N0 | 28(93.3) |
| N1 | 2(6.7) |
| **Distant metastasis** | |
| M0 | 30(100) |
| M1 | 0(0) |
| **Edmondson-steiner grades** | |
| I | 2(6.7) |
| II | 15(50) |
| III | 13(43.3) |
| IV | 0(0) |
